# A global call for action to include gender in research impact assessment

**DOI:** 10.1186/s12961-016-0126-z

**Published:** 2016-07-19

**Authors:** Pavel V. Ovseiko, Trisha Greenhalgh, Paula Adam, Jonathan Grant, Saba Hinrichs-Krapels, Kathryn E. Graham, Pamela A. Valentine, Omar Sued, Omar F. Boukhris, Nada M. Al Olaqi, Idrees S. Al Rahbi, Anne-Maree Dowd, Sara Bice, Tamika L. Heiden, Michael D. Fischer, Sue Dopson, Robyn Norton, Alexandra Pollitt, Steven Wooding, Gert V. Balling, Ulla Jakobsen, Ellen Kuhlmann, Ineke Klinge, Linda H. Pololi, Reshma Jagsi, Helen Lawton Smith, Henry Etzkowitz, Mathias W. Nielsen, Carme Carrion, Maite Solans‐Domènech, Esther Vizcaino, Lin Naing, Quentin H. N. Cheok, Baerbel Eckelmann, Moses C. Simuyemba, Temwa Msiska, Giovanna Declich, Laurel D. Edmunds, Vasiliki Kiparoglou, Alison M. J. Buchan, Catherine Williamson, Graham M. Lord, Keith M. Channon, Rebecca Surender, Alastair M. Buchan

**Affiliations:** Medical Sciences Division, University of Oxford, John Radcliffe Hospital, Oxford, OX3 9DU United Kingdom; Nuffield Department of Primary Care Health Sciences, University of Oxford, Radcliffe Primary Care Building, Woodstock Road, Oxford, OX2 6GG United Kingdom; Agency for Health Quality and Assessment of Catalonia (AQuAS), Carrer de Roc Boronat, 81, ES-08005 Barcelona, Spain; The Policy Institute, King’s College London, Strand Campus, London, WC2R 2LS United Kingdom; Alberta Innovates – Health Solutions, 10104-103 Avenue NW, Edmonton, AB T5J 4A7 Canada; Fundación Huésped, Pasaje A. Peluffo 3932 (C1202ABB), Buenos Aires, Argentina; Qatar National Research Fund, P.O. Box 5825, Doha, Qatar; Qatar Foundation, P.O. Box 5825, Doha, Qatar; Department of Studies and Planning, The Research Council, P.O. Box 1422, Al Azaiba, 130 Oman; Commonwealth Scientific and Industrial Research Organisation, P.O. Box 883, Kenmore, Brisbane, 4069 Australia; Melbourne School of Government, The University of Melbourne, Parkville, Victoria 3010 Australia; School of Population Health, University of Western Australia, Perth, WA 6009 Australia; Knowledge Translation Australia Pty Ltd., Melbourne, Victoria Australia; Faculty of Business and Economics, University of Melbourne, 198 Berkeley Street, Parkville, Victoria 3010 Australia; Saïd Business School, University of Oxford, Park End Street, Oxford, OX1 1HR United Kingdom; The George Institute for Global Health, University of Oxford, 34 Broad Street, Oxford, OX1 3BD United Kingdom; The George Institute for Global Health, University of Sydney, P.O. Box M201, Missenden Road, Sydney, NSW 2050 Australia; RAND Europe, Westbrook Centre, Milton Road, Cambridge, CB4 1YG United Kingdom; Novo Nordisk Foundation, Tuborg Havnevej 19, DK-2900 Hellerup, Denmark; Lundbeck Foundation, Scherfigsvej 7, DK-2100 Copenhagen, Denmark; Institute for Economics, Labour and Culture, Goethe-University Frankfurt, Senckenberganlage 31, 60325 Frankfurt am Main, Germany; Medical Management Centre, Department of Learning, Informatics, Management and Ethics (LIME), Karolinska Institutet, Tomtebodavaegen 18a, 171 77 Stockholm, Sweden; Horizon 2020 Advisory Group for Gender, European Commission, Brussels, Belgium; National Initiative on Gender, Culture and Leadership in Medicine: C-Change, Brandeis University Women’s Studies Research Center, 415 South Street, MS 079, Waltham, MA 02454 United States of America; Department of Radiation Oncology, Center for Bioethics and Social Sciences in Medicine, University of Michigan, Ann Arbor, MI 48109 United States of America; Department of Management, Birkbeck, University of London, Malet Street, London, WC1E 7HX United Kingdom; International Triple Helix Institute, 1520 Sand Hill Road, A210, Palo Alto, CA 94304 United States of America; Gendered Innovations, History Department, Stanford University, 450 Serra Mall, Stanford, CA 94305 United States of America; Health Sciences Department, Universitat Oberta de Catalunya, Av. Tibidabo 39-43, ES-08035 Barcelona, Spain; PAPRSB Institute of Health Sciences, Universiti Brunei Darussalam, Jalan Tungku Link, Gadong, BE1410 Brunei Darussalam; Faculty of Integrated Technologies, Universiti Brunei Darussalam, Jalan Tungku Link, Gadong, BE1410 Brunei Darussalam; QS Intelligence Unit, Quacquarelli Symonds Ltd, 4 Heathgate, Agincourt Rd, London, NW3 2NT United Kingdom; Department of Public Health, School of Medicine, University of Zambia, Nationalist Rd, Lusaka, Zambia; Research Support Centre, College of Medicine, University of Malawi, P.O. Box 360, Chichiri, Blantyre 3 Malawi; Assembly of Women for Development and the Struggle against Social Exclusion (ASDO), via Guido Reni 56, 00196 Rome, Italy; NIHR Oxford Biomedical Research Centre, Joint Research Office, Churchill Hospital, Oxford, OX3 7LE United Kingdom; Oxford University Hospitals NHS Foundation Trust, John Radcliffe Hospital, Oxford, OX3 9DU United Kingdom; Department of Physiology, University of Toronto, 1 King’s College Circle, Toronto, Ontario M5S 1A8 Canada; Women’s Health Academic Centre, King’s College London, Guy’s Hospital, London, SE1 1UL United Kingdom; NIHR Biomedical Research Centre at Guy’s and St Thomas’ NHS Foundation Trust and King’s College London, Guy’s Hospital, London, SE1 9RT United Kingdom; Guy’s and St Thomas’ NHS Foundation Trust, Guy’s Hospital, London, SE1 9RT United Kingdom; MRC Centre for Transplantation, King’s College London, Guys’ Hospital, London, SE1 9RT United Kingdom; Department of Social Policy and Intervention, University of Oxford, Barnett House, 32-37 Wellington Square, Oxford, OX1 2ER United Kingdom; Institute of Social and Economic Research, Rhodes University, P.O. Box 94, Grahamstown, 6140 South Africa

**Keywords:** Research impact assessment, Gender, Path dependency, Health research, Science policy, Athena SWAN, Call for action

## Abstract

Global investment in biomedical research has grown significantly over the last decades, reaching approximately a quarter of a trillion US dollars in 2010. However, not all of this investment is distributed evenly by gender. It follows, arguably, that scarce research resources may not be optimally invested (by either not supporting the best science or by failing to investigate topics that benefit women and men equitably). Women across the world tend to be significantly underrepresented in research both as researchers and research participants, receive less research funding, and appear less frequently than men as authors on research publications. There is also some evidence that women are relatively disadvantaged as the beneficiaries of research, in terms of its health, societal and economic impacts. Historical gender biases may have created a path dependency that means that the research system and the impacts of research are biased towards male researchers and male beneficiaries, making it inherently difficult (though not impossible) to eliminate gender bias. In this commentary, we – a group of scholars and practitioners from Africa, America, Asia and Europe – argue that gender-sensitive research impact assessment could become a force for good in moving science policy and practice towards gender equity. Research impact assessment is the multidisciplinary field of scientific inquiry that examines the research process to maximise scientific, societal and economic returns on investment in research. It encompasses many theoretical and methodological approaches that can be used to investigate gender bias and recommend actions for change to maximise research impact. We offer a set of recommendations to research funders, research institutions and research evaluators who conduct impact assessment on how to include and strengthen analysis of gender equity in research impact assessment and issue a global call for action.

## Gender bias in health research

Global investment in biomedical research has grown significantly over recent decades. In 2010, global investment reached US$240 billion (adjusted for purchasing power parity), delivering important health dividends to patients and citizens [1]. However, not all of this investment is distributed evenly by gender. It follows, arguably, that scarce research resources may not be optimally invested by either not supporting the best science or by failing to investigate topics that benefit women and men equitably. Gender bias in biomedical and health research involves both biological sex differences and sociocultural differences in the way women and men behave, and in the way they are treated [2]. There is evidence that gender bias in biomedical and health research can occur at all stages of the research process across the following four domains.

First, women tend to be significantly underrepresented in research both as researchers and research participants. Although in 2013, women had reached 55% of admissions to medical schools in the United Kingdom and 47% in the United States of America, they constituted only 28% of faculty physician-scientists in the United Kingdom and 38% in the United States [3]. Moreover, female faculty members tend to report less favourable experiences and feel excluded [4, 5]. Women are also underrepresented as research participants. Historical analysis demonstrated a male bias in biomedical research throughout the 20th century: it was evident in 8 of the 10 biomedical fields surveyed in 2009 [6]. For example, while women represent nearly half of people living with HIV, they are under-represented in clinical studies of HIV antiretroviral drugs (19%), prophylactic vaccines (38%) and curative strategies (11%) [7]. Women are also under-represented in high-impact studies of non-sex-specific cancers [8].

Second, female investigators tend to receive less research funding than their male counterparts in absolute and relative terms. This may occur because there are fewer women investigators who apply for research funding [9], and those who apply receive smaller awards than men [10]. There is some evidence that, in certain settings, this is also amplified by reported nepotism and sexism in peer-review [11]. Although an earlier meta-analysis of empirical studies from different fields concluded that women applying for grants have statistically significant lower odds of receiving funding than men by approximately 7% [12], a more recent and methodologically advanced meta-analysis of the same data [13] and a recent empirical study [14] concluded the contrary. United States research suggests that female early career researchers receive significantly less start-up support from their institutions [15] and are significantly less likely than men to achieve independent funding awards [16]. Further, United States research suggests that women at particular career stages are less likely to apply for the competitive grants for which they are eligible, compared to their male counterparts [17]. Research from the Netherlands demonstrated gender bias favouring male grant funding applicants in the evaluation of the ‘quality of researcher’, but not the ‘quality of proposal’ [18]. Even in the fields where there is no difference in funding rates between the genders, such as radiology, women have less total grant funding than men [19].

Third, women tend to appear less frequently than men as winners of prestigious scientific awards and as authors of research publications. Among 210 Nobel Laureates in Physiology or Medicine awarded from 1901 to 2015, there are only 12 (5.7%) women [20]. Despite significant progress in recent decades, women are still underrepresented as authors of research articles in medical journals [21, 22], especially as first and senior authors [23, 24]. For example, the proportion of women first authors in six prominent international medical journals increased from 6% in 1970 to 29% in 2004, and the proportion of women senior authors increased during the same period from 4% to 19% [23]. In radiology, the proportion of women first authors increased from 8% in 1978 to 32% in 2013 [25]. The proportion of women first authors in high impact general medical journals increased from 27% in 1994 to 37% in 2014, but it has recently plateaued and even declined in some journals [26].

Finally, women may be disadvantaged as the beneficiaries of research in terms of its health, societal and economic impacts [27–29]. There is evidence to suggest that research that does not account for gender differences can result in inaccurate conclusions about how women respond to disease and this, in turn, will influence the effectiveness of treatment choices [30, 31]. For example, ‘Yentl syndrome’ describes sex bias in the management of coronary heart disease due to the fact that medical research had predominantly studied symptoms of heart attacks in men [32]. Historically, those women who presented with symptoms of heart attack similar to those in men received the same diagnostic and treatment procedures as men, but those whose symptoms presented differently were not properly treated and may have died unnecessarily. For such reasons, the League of European Research Universities has recently stressed that, without including gender analysis in research, the impact of science may not be equally beneficial for both men and women [33].

In what follows, we elaborate on the path dependent nature of gender bias in science and why it is difficult, but not impossible to address. Second, we outline key characteristics of research impact assessment; we argue that by investigating gender bias, research impact assessment can become a force for good in moving science policy and practice towards gender equity. Third, we offer a set of recommendations about how research funders, research institutions and research evaluators can include and strengthen analysis of gender equity in research impact assessment. We conclude by issuing a global call for action.

## Path dependency of gender bias

Historical gender biases have generated a form of path dependency such that the research system, including research impacts, is, to some extent, male oriented. In most countries, women began entering higher education institutions in substantial numbers only from the beginning of the 20th century. However, over the last century, remarkable social progress has been made, across much of the globe, towards gender equity. Proponents of the so-called ‘pipeline’ argument believed that once numbers of women entering universities reached sufficient numbers and they were not discriminated against for admission into the pipeline, gender bias in the representation of women in science would gradually disappear [34–38]. Indeed, in many countries where there are strict non-discrimination laws, women have reached 40% or more in admissions to medicine and other university health science degrees – a figure that is sometimes used as a threshold for gender balance [39]. Nevertheless, gender bias in health research still persists because the path dependent nature of science makes it difficult to change the status quo.

The notion of path dependency, which originates in economics and political science, suggests that our current actions depend on existing knowledge and past decisions, and therefore strong conjunctural forces are required to move policy and practice away from the established path [40]. Research on path dependency in political science suggests that such forces are associated with rational values and a strong centralised authority such as governments wishing to change current policy and practice [40, 41]. Path dependent gender bias in research is hard to address because it is often institutionalised as policies, practices, beliefs and written or unwritten rules of behaviour that structure modern science and society.

The path dependent tendency of science is aptly illustrated by Isaac Newton’s metaphor of ‘standing on the shoulders of giants’. Historically, these giants of science have been overwhelmingly male [42]. The ‘Matthew Matilda effect’ highlights the tendency for women’s work to be systematically omitted in the history of scientific achievements or even to be misattributed to men [43]. This is part of a wider historical tendency within a range of societies (regardless of culture, religion or political organisation) for an unequal distribution of scientific resources and power between men and women. Both the current body of scientific knowledge and current scientific practices are consequently shaped predominantly by male perceptions and norms, which are often influenced by both conscious and unconscious gender bias.

As far as scientific knowledge is concerned, there has been a propensity not to take into consideration possible sex and gender differences in the research design and analysis of clinical trials [44, 45]. However, given that both the occurrence and outcomes of a range of medical conditions differ for men and women, a large body of clinical knowledge which does not take relevant sex and gender differences into account may be flawed [44]. For example, 50 years ago, few studies included women in prospective cohort studies of all-cause or coronary heart disease mortality [46]. Although a growing number of peer-reviewed health research journals now have editorial policies requiring sex- or gender-specific reporting of results [29], there remain many journals without such policies [47].

Scientific practices and structures can also be biased against women. Male and female leaders may have different leadership traits: women are more likely to focus on collaboration and may have less interest, and fare less well, in hierarchical organisation [48, 49]. However, appointment criteria for scientific leadership are often based on classical ‘male-gendered’ traits [50–53]. Academic promotion and tenure criteria have also traditionally been based on a male-gendered career trajectory which collides with the biological clock of women wishing to raise families [54], although much progress has been made in some countries to protect against bias towards those taking career breaks. Implicit bias affects the advancement and promotion of women within the research workforce [55], while recruitment materials may be interpreted through a gender bias lens, with recommendation letters for men being read as stronger than those for women with equal qualifications [56]. Likewise, women candidates for tenured research positions are sometimes put at a disadvantage due to ‘backdoor hiring’ practices and mobilisations of informal, potentially gendered, network ties, in academic recruitment and selection [57, 58].

Nevertheless, there is an opportunity to move science policy and practice towards greater gender equity, because scientific enterprise is founded on rational values. In essence, scientific enterprise strives to produce objective knowledge without bias, with a view to bringing societal and economic benefits to humanity. Greater gender equity helps scientific enterprise achieve objective knowledge regardless of the ways objectivity is conceived of by different epistemological communities. The traditional view is that objective knowledge exists and can be achieved by removing different biases, including the gender bias. This view is questioned by feminist scholars who contend that all knowledge is gender-biased in one way or another and thus objectivity can be achieved by increasing the pluralism of perspectives to balance biases and by critically evaluating biases in both knowledge and methods of enquiry [59–62].

**Fig. 1:**
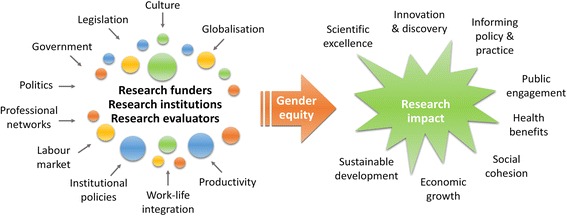
The gender equity pathway to maximise research impact. Shown are the forces that influence the key stakeholders in promoting gender equity to maximise different possible types of research impact

Although the value of gender equity for increasing the objectivity of science is acknowledged by different epistemological communities, the scope for centralised policy action to maximise research impact through the gender equity pathway within scientific enterprise is limited. This is partly because many research funders and institutions are decentralised (and cross multiple sectors including private, public and third-sector), and partly because changes in policy may reduce but not eliminate gender bias. Whilst national and local policies may be in place, the reality of research enterprise is that decision-making authority is devolved to the level of departments, centres and, sometimes, individual principal investigators. Moreover, scientific enterprise has (encouragingly) become globalised (there is increased mobility of researchers, and many research funders and institutions seek to become globally competitive in the knowledge economy). It follows that gender equity in research requires action at a global level to be maximally effective (Fig. 1).

## Research impact assessment

Research impact assessment is the multidisciplinary field of scientific inquiry that examines the research process to maximise scientific, societal and economic returns on investment in research. Unlike the primary orientation of basic science, which is concerned with the advancement of knowledge for its own sake, research impact assessment (a form of research on research) is predominantly oriented towards applied research objectives of influencing policy and practice. Research impact assessment has developed its own methods of analysis, drawing on other disciplines and fields of knowledge [63–68]. Its combination of applied research objectives and scientific rigour should allow research impact assessment to become a force for good in moving science policy and practice towards gender equity by investigating gender bias and recommending actions for change.

**Fig. 2:**
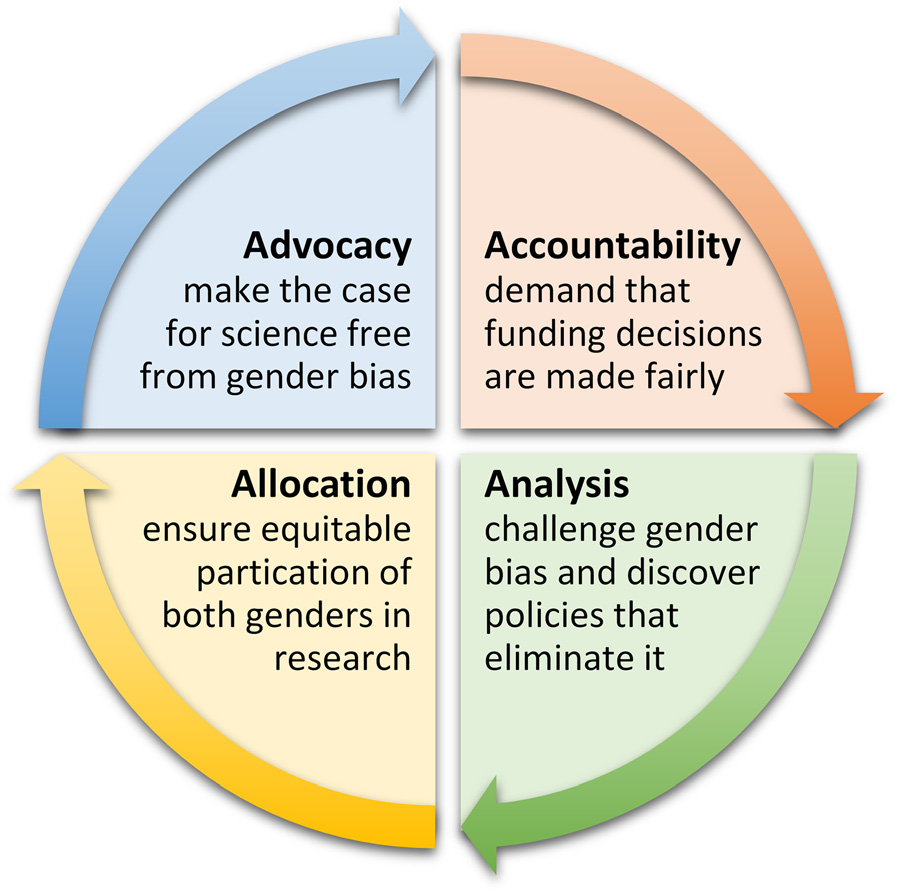
The Four “As” of research impact assessment with regard to gender equity

The driving forces behind research impact assessment have been conceptualised into the Four “As” of Advocacy, Accountability, Analysis and Allocation [69]. Given the challenges of addressing gender bias in research, each of the Four “As” of research impact assessment are necessary to address gender bias in research (Fig. 2):

- Advocacy is needed to ‘make the case’ [69] for science free from gender bias, by highlighting the scientific, societal, and economic benefits of gender equity in research.
- Accountability to the public requires that funding and staffing decisions by research funders and research institutions are made fairly, and that an account of such decision-making is captured and reported transparently through appropriate metrics.
- Analysis is necessary to challenge gender bias and discover policies that eliminate it, as well as conditions under which these policies could be transferred to other organisations and countries.
- Allocation of research funding to ensure equitable participation of both genders in research is imperative for the legitimisation of and public support for science [38].

Various methodological approaches may be used within research impact assessment to investigate and address gender bias (Table 1). For example, logic modelling and related theories of change [70] can be applied to systematically investigate gender equity at all stages of the research process, as well as effective mechanisms for ensuring the transparent and equitable distribution of resources and power. Bibliometrics and other traditional research output metrics (scientometrics) can be used to measure gender-based differences in research outputs, their academic impacts, including gender-based citation behaviour, and non-academic impacts [23, 71–74]; however, unless supplemented by additional methodologies, they will not illuminate the reasons for such differences. Potentially, the more immediate academic and non-academic impact of research can also be measured using non-traditional web-based metrics (altmetrics) [75, 76]. Surveys and narrative case studies can be especially useful in investigating these differences. Curriculum vitae and survey data can be combined to measure variations in researchers’ societal outreach and orientation [77], and text-mining algorithms can be employed to analyse developments in the scholarly attention to gender and sex issues in biomedical research [78]. Information and management science approaches can be employed to develop performance management systems (such as the recently described ‘balanced scorecard’ [79]) for assessing and monitoring gender equity both in the awarding of grants and selection of research topics. Finally, economic modelling and cost-benefit analysis could be employed to examine how gender equity can maximise economic returns on investment in research.

**Table 1:** Selection of methods in research impact assessment and how they may be used to investigate and address gender bias

Methods	Applications
Logic modelling describes and graphically represents the chains of results associated with all stages of the research process; theories of change are formulated on the basis of these chains of results to explain how to achieve the desired results	To provide a framework to systematically investigate gender equity at all stages of the research process, and investigate how gender equity can help maximise desired research outputs and impacts
Scientometrics pertain to the statistical analysis of articles and citations in academic journals (bibliometrics) and other research-based outputs, e.g. patents, commercialisation	To measure gender-based differences in research outputs, their academic impacts, including gender-biased citation behaviour, and non-academic impacts
Altmetrics measure the online attention to academic articles on social media, mainstream news websites, blogs and social bookmarking websites	To measure gender-based differences in more immediate academic and non-academic impact of research
Surveys, curriculum vitae data, narrative case studies and text-mining algorithms collect quantitative and qualitative information that may not already have been captured as part of the research process	To investigate gender-based differences in perceptions of and approaches to the research process, outcomes and impacts
Information and management science approaches allow developing balanced scorecards and other performance management systems	To assess and monitor gender equity in research organisations
Economic modelling categorises and cost-benefit analysis assesses different types of benefits from research	To examine how gender equity can maximise economic returns on investment in research

Whichever methodological approaches are employed, active engagement with users of impact assessment is needed, in turn, to disseminate findings and influence policy and practice. Communication of these findings is likely to be strengthened by the use of infographics, diagrams, charts and other visual tools to help clearly convey qualitative and quantitative data and trends. For example, recent analysis of the United Kingdom’s Research Excellence Framework 2014 impact case studies has used innovative infographics, alluvial and chord diagrams, word clouds, heat maps and impact wheels, synthesising complex data to reveal where research has had a societal impact [80]. With increasing use of the web and social media by researchers and research users, impact assessment results can be rapidly communicated through a variety of media, including research blogs, social networks and web feeds. Gender-sensitive visualisation and dissemination can further enhance engagement with users of impact assessment.

## Recommendations

For research impact assessment to become a force for good in moving science policy and practice towards gender equity, we propose that gender be routinely included in research impact assessment. Based on our knowledge and experience of the field, we – a group of scholars and practitioners representing research leaders from Africa, America, Asia and Europe – offer recommendations to research funders, research institutions and research evaluators (i.e. those who conduct research impact assessment) on how to include and strengthen analysis of gender equity in research impact assessment. Our recommendations are outlined below and summarised in Table 2.

**Table 2:** Recommendations to include and strengthen analysis of gender equity in research impact assessment

Stakeholders	Recommendations
Research funders	• Conduct retrospective and prospective observational gender-based research impact assessment to inform implementation of gender equity policies • Adopt policies to ensure that researchers address relevant sex and gender issues in their research designs and analyses • Identify and apply evidence-based approaches to integrating gender equity into research funding criteria • Where appropriate, support theoretical and applied research on the scientific, societal and economic impact of gender equity in research
Research institutions	• Establish information systems for collecting and analysing gender-based information on the research process • Work towards improving and refining the quality of gender-based data • Train and support interested students and staff to include gender in research impact assessment • Establish gender equity activity as a criterion for performance management and annual appraisal and promotion
Research evaluators	• Engage with the research impact assessment literature with a view to identifying and applying evidence-based tools and approaches to gender-sensitive research impact assessment • Identify, investigate and address gender differences in research production and impact, including commercialisation • Increase the objectivity of research impact assessment by conducting evaluations in gender-balanced teams using an appropriate mix of methods • Collaborate and share knowledge through gender-balanced networks and communities of practice • Engage impact assessment users and the public in debate on gender bias and recommend actions for change

## Research funders

Internationally, research funders have long conducted research impact assessment in order to demonstrate accountability to taxpayers and philanthropists as well as to inform their resource allocation practices. In so doing, many funders already have established systems for collecting basic gender-based information on the research they fund. Such information is often required to demonstrate compliance with anti-discrimination legislation. However, it is not routinely used for research impact assessment. We recommend that research funders conduct retrospective research impact assessment using gender-based information already available. We suggest this should inform the development and implementation of gender equity policies, and lead to more gender-sensitive prospective data collection. This would help identify research programmes and practices that are most reflective of gender equity, areas which may need may need further exploration or development, and policies to support fairer application processes. For example, the Australian Research Council has recently introduced a ‘gender equity action plan’, including policies to report gender disaggregated data and monitor the gender outcomes of selection rounds [81].

It has long been recognised that research results may not equally apply to men and women if scientific evidence fails to consider relevant sex and gender issues. However, developing formal policies and approaches by research funders to encourage researchers to include considerations of sex and gender in their research designs and analyses is a relatively recent phenomenon [82]. For example, since 2009, the Canadian Institutes of Health Research requires that all grant applicants consider whether their research designs include sex and gender when appropriate [83]. In 2014, the United States National Institutes of Health announced new supplemental awards to explore the effects of sex and gender in preclinical and clinical studies [84]. The European Commission has integrated gender- and sex-based analysis into Horizon 2020 – its biggest research and innovation programme for 2014–2020 [85]. Such policies encourage researchers to study both sexes to aid scientific innovation and better health for all citizens. We recommend that research funders adopt policies to ensure that researchers address relevant sex and gender issues in their research designs and analyses.

There are also research funders that now require research institutions applying for research funding to demonstrate how they support gender equality in research careers. This is mainly achieved through various award schemes designed to promote structural change in research institutions [86]. For example, the United Kingdom National Institute for Health Research made designation and funding of its Biomedical Research Centres conditional upon the achievement by academic applicants of at least the Silver Award of the Athena Project and the Scientific Women’s Academic Network (SWAN) Charter for Women in Science, which recognises work undertaken by research institutions to advance women’s careers in science, technology, engineering, mathematics and medicine (STEMM) [79]. Another example of integrating gender equity into research funding criteria is the report card developed by the New York Stem Cell Foundation to assess institutions in grant application processes [87]. We recommend that research funders identify and apply evidence-based approaches to integrating gender equity into research funding criteria.

So far, relatively little scholarly attention has been given to the scientific, societal and economic impact of gender equity in research. Debate on gender equity in research has been primarily driven by human rights and equality imperatives and is based on evidence that is sparse and of variable methodological quality [3]. Importantly, experimental research shows gender differences in how the quality of evidence revealing gender bias is evaluated by women and men [88]. There is a need to systematise the current evidence to map more precisely the state of knowledge, ignorance and uncertainty in the field, and identify gaps in the evidence base where new research is needed. Such a scoping exercise is likely to highlight the need for further comparative effectiveness research to determine the most effective policy interventions, as well as the conditions under which particular interventions can be effective in different settings. Additionally, fields beyond medicine (such as higher education studies and the sociology of science) may provide important theoretical perspectives that will help guide the design of empirical studies. We recommend that, where appropriate, research funders support theoretical and applied research on the scientific, societal and economic impact of gender equity in research.

## Research institutions

Increasingly, research institutions realise that “*winning the talent war for women*” [89] has great potential to contribute to their international competitiveness and future growth. However, research institutions do not always have information systems that allow disaggregating key research performance data by gender. Moreover, progress towards gender equity and the enhanced ability to compete for the best students, faculty and staff of both genders are not routinely considered as key performance indicators by universities themselves and as a basis for competition with their peers globally, e.g. in university rankings and league tables. We recommend that universities and other research institutions establish systems for collecting and analysing gender-based information on the research process.

Gender equity in research institutions tends to vary by level of seniority, department, and discipline. Women tend to be better represented at entry and junior levels and underrepresented at senior levels, especially in the STEMM sciences and clinical trials. If gender analysis is conducted on too coarse a unit of aggregation, different biases can inadvertently cancel each other out (e.g. women in nursing, men in surgery). We recommend that research institutions work towards improving and refining the quality of gender-based data, e.g. by ensuring that it is possible to disaggregate gender-based information vertically by the level of seniority and horizontally by department and discipline.

Research institutions have a unique role to play in the inclusion of gender in research impact assessment because they educate and employ today’s and tomorrow’s research evaluators. Research institutions can create awareness amongst their students and staff about the importance of gender analysis and provide them with the necessary knowledge and skills to analyse and report gender-specific and gender-comparative results. For example, they can include elements of gender analysis and social science perspectives on gender biases in knowledge and methods of enquiry in the teaching curricula of the relevant disciplines; organise multi-disciplinary workshops and seminars to develop the competencies of the students and staff interested in research impact assessment; implement comprehensive diversity and inclusion plans [90]; consider targets and quotas for women in leadership [91]; actively develop expert knowledge leadership in gender-sensitive research impact [92]; and even evaluate and address the gender pay gap [93, 94]. We recommend that research institutions train and support interested students and staff to include gender in research impact assessment.

Most research institutions across the globe now have in place programmes and initiatives to support gender equity, either as part of their own efforts or as a response to the incentives provided by research funders. For example, most research-intensive universities in the United Kingdom participate in the Athena SWAN Charter for Women in Science and its national and regional equality networks [95]. This requires extensive data collection and analysis, discussions in working groups and committees, and preparation and implementation of action plans. Female faculty bear much of the burden for preparing Athena SWAN award applications [87], which may even be to the detriment of their core scientific activity and thus productivity and promotion. Therefore, it is important to ensure that gender equity activity undertaken by faculty is recognised within their own institutions and scientific fields. We recommend that research institutions include gender equity activity as a criterion for performance management and annual appraisal and promotion.

## Research evaluators

The science of research impact assessment has already developed a number of validated evidence-based approaches that can be used for gender analysis. Moreover, there is an emerging evidence base of successful research impact assessment studies focussing specifically on gender [96, 97]. By using similar tools in new studies, research evaluators can test their robustness and extend the evidence base in ways that allow synthesis across studies. Therefore, we recommend that research evaluators engage with the research impact assessment literature with a view to identifying and applying evidence-based tools and approaches to gender-sensitive research impact assessment. However, when new kinds of research questions are being asked about gender, it may be appropriate to develop new tools and approaches.

Gender analysis can reveal important differences between men and women in research production and impact that can inform policies to optimise performance of the scientific enterprise. Whereas male scientists may be more productive during the early stages of their careers, the productivity rates of female scientists may equal or surpass those of men later in their careers [98, 99]. Whereas male physician-scientists may be more interested in basic science with long-term impacts, female physician-scientists may be more interested in clinical work with more immediate patient benefits [100]. Likewise, there may be important gender differences in co-authorship behaviour, interdisciplinary collaboration and societal impact [99]. Whereas women leaders may be more collaborative, more nurturing, and share power and uncertainty more, male leaders may be more hierarchical; and whereas male researchers may be geared towards scientific rewards and recognition, female researchers may have a greater societal orientation and output [77]. However, on the basis of the evidence in relation to the quality of women’s academic outputs and citations, “*there is no evidence that women do less important work than men*” [52]. Thus, where gender differences exist, this not only requires further exploration, but also highlights the need for the use of appropriate metrics that do not lead to a systematic bias in assessing the research production and impact of either men or women. We recommend that research evaluators investigate gender differences in research production and impact, especially in terms of productivity, collaboration, impact time lags and types of impact, and carefully select appropriate metrics to avoid bias.

It is important to ensure that the act of research impact assessment itself does not become affected by gender bias. For example, there may be a gender bias in the selection of methods and models of inquiry. Whereas quantitative methods and a positivist natural science model of inquiry have been traditionally associated with male researchers, qualitative methods and an interpretivist social science model of inquiry have been traditionally associated with female researchers [101]. Moreover, there is a disagreement between different epistemological communities as to whether the objectivity of knowledge can be achieved by removing gender bias or by increasing the pluralism of perspectives to balance different gender biases [60]. Regardless of the ways objectivity is conceived of by different epistemological communities, increasing gender equity in research teams and the pluralism of methods can increase the objectivity of knowledge. We recommend that research evaluators increase the objectivity of research impact assessment by conducting evaluations in gender-balanced teams using an appropriate mix of methods.

Research impact assessment is a rapidly growing field of knowledge spanning disciplinary, organisational and geographical boundaries. Research evaluators can stay abreast of new knowledge and strategic developments in the field through collaboration and knowledge sharing. These can also help identify solutions to common problems and provide feedback without wasting time and resources. Moreover, participation in boundary-spanning networks and communities of practice can diffuse innovative ideas and unleash collective creativity to discover new approaches for the greater good. In doing so it is important that networks and communities of practice strive to achieve gender balance among their members and leaders, and routinely address gender issues. We recommend that research evaluators collaborate and share knowledge through gender-balanced networks and communities of practice.

Research evaluators have a potentially transformative role to play in the scientific enterprise because research impact assessment is predominantly oriented towards influencing policy and practice. As research evaluators investigate research outputs and outcomes, they engage with a wide range of stakeholders and communicate their findings to decision-makers. This gives research evaluators an opportunity not only to investigate gender bias, but also to suggest a course of policy interventions to promote gender equity. The knowledge of science policy in various settings also enables research evaluators to initiate public debate and advocate for science free from gender bias. We recommend that research evaluators engage impact assessment users and the public in debate on gender bias and recommend actions for change.

## Conclusions

Growing global investment in biomedical research is unlikely to result in outstanding science that benefits women and men equitably if current levels of conscious and unconscious gender bias in health research persist. Gender bias is difficult to eliminate, in part because of the historical late entry of women into higher education and research, and a tendency of path dependency in science. However, we argue that research impact assessment can become a force for good in moving science policy and practice towards gender equity by revealing and challenging gender bias. Success in applying research impact assessment to address gender bias will depend on sustained action by multiple stakeholders at all stages of the research process and internationally. We call on research funders, research institutions and research evaluators globally to include gender in research impact assessment in order to maximise scientific, societal and economic returns on investment in research.

## Abbreviations

Athena SWAN, Athena Project and the Scientific Women’s Academic Network; STEMM, science, technology, engineering, mathematics and medicine
